# Electron Transfer-Based Single Molecule Fluorescence as a Probe for Nano-Environment Dynamics

**DOI:** 10.3390/s140202449

**Published:** 2014-02-03

**Authors:** Ruiyun Chen, Ruixiang Wu, Guofeng Zhang, Yan Gao, Liantuan Xiao, Suotang Jia

**Affiliations:** State Key Laboratory of Quantum Optics and Quantum Optics Devices, Institute of Laser Spectroscopy, Shanxi University, Taiyuan 030006, China; E-Mails: chenry421@163.com (R.C.); Wurx464628021@163.com (R.W.); guofeng.zhang@sxu.edu.cn (G.Z.); ggnnool@163.com (Y.G.); tjia@sxu.edu.cn (S.J.)

**Keywords:** single-molecule probes, electron transfer, dynamics, nano-environments

## Abstract

Electron transfer (ET) is one of the most important elementary processes that takes place in fundamental aspects of biology, chemistry, and physics. In this review, we discuss recent research on single molecule probes based on ET. We review some applications, including the dynamics of glass-forming systems, surface binding events, interfacial ET on semiconductors, and the external field-induced dynamics of polymers. All these examples show that the ET-induced changes of fluorescence trajectory and lifetime of single molecules can be used to sensitively probe the surrounding nano-environments.

## 1. Introduction

Nanoscience, which is concerned with the dynamic, structure and properties of materials with dimensions ranging from a few nanometers to less than 100 nanometers, has attracted considerable interest in recent years. Although the ensemble average technique is a powerful tool to extract averaged information, the dynamics down to the molecular scale remain obscured due to the ensemble averaging. Moreover, at the nanometer scale most materials display heterogeneity properties which cannot be revealed by ensemble techniques. In the past two decades, single-molecule spectroscopy has proven to be a powerful approach for studying inhomogeneous dynamics in complex systems where disorder is intrinsic [1–9]. A variety of optical experiments have been designed and performed to allow the detection of single molecules in different environments, including solids [10,11], surfaces [12,13] and supercooled liquids [14,15], *etc*. Single-molecule detection completely removes the ensemble averaging, making it possible to directly observe the spatial heterogeneity on the nanometer length scale. Single molecules can serve as sensitive nano-probes if the properties of the emitted fluorescence can be traced back to the change(s) in their environment. Numerous tools based on the detection of the fluorescence characteristics of single molecules, involving polarization [16–20], spectrum [21,22], and lifetime [23,24], have been proposed to measure nano-environment dynamics.

Electron transfer (ET) [25,26] is a fundamental physical process both in natural and artificial systems, which occurs when an electron moves from one chemical species to another chemical species. ET introduces changes in the fluorescence intermittency and the fluorescence lifetime of single molecules. Fluorescence intermittency, or blinking, is the phenomenon of random switching between the “on” (bright) state and the “off” (dark) state of a single molecule under continuous excitation. Short off-duration-time blinking events lasting on the order of microseconds to a few milliseconds are recognized in single molecule experiments [27] and have been found to be attributable to the intersystem crossing from a singlet excited state to a triplet dark state. This mechanism results in single-exponential distributions of on- and off-times. However, fluorescence blinking with long duration times has been found in a study of Zondervan *et al.* [28,29]. The long off-duration-times are unlikely due to triplet state. Nowadays, most researchers follow the hypothesis by Efros and Rosen that the central role in blinking is ET and the dark state is ionized [30]. It is generally admitted that the long off-time blinking of fluorescence arises from charge separation from the excited state to a nearby acceptor. It has been proved that ET processes are responsible for fluorescence blinking on the subsecond to several seconds time scale of single molecule electron donor-acceptor systems and single-molecule to semiconductor electron donor systems. Clifford *et al.* investigated the local environment dependence of the blinking behavior of the organic dye Atto647N in various polymer matrixes [31]. They proposed that the blinking can be attributed to dye radical ions formed by photoinduced ET from or to the environment, which suggests that the blinking behavior of a single organic molecule can be a sensitive probe of its immediate environment. Fluorescence lifetime is another ET-induced characteristic of single molecules, which is an intrinsic property of a fluorophore and does not dependent on the wavelength of excitation, duration of light exposure, one or multiphoton excitation, and the method of measurement. Observed fluctuations of the fluorescence lifetimes of single molecules have been attributed to ET behavior from (or to) the local environments. ET is highly distance-dependent and this has been observed through the measurements of fluorescence lifetime for single molecules [32]. Fluorescence lifetime fluctuation of single flavin molecule arising from an excited state ET has been used to probe protein conformational dynamics [33].

In this paper, we review the main uses of single molecules in studies of nano-environment dynamics, by measuring the change of fluorescence trajectory and lifetime induced by ET. We focus on some applications, including dynamics of glass-forming systems, surface binding events, ET between single dye molecules and semiconductors, and the external field-induced dynamics of polymers.

## 2. Principles and Techniques

### 2.1. Physical Principles

**Figure 1.**
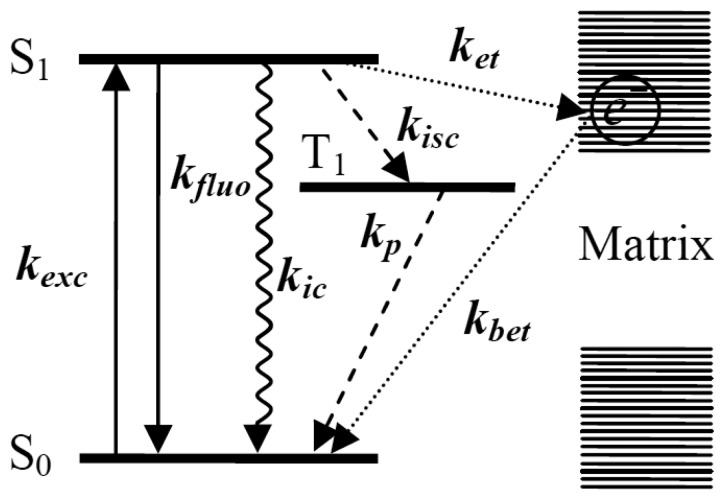
Scheme of single molecule energy levels and the electron transfer (ET) between a single molecule and the matrix. *S*_0_, *S*_1_ and *T*_1_ represent the ground state, excited state and triplet state of single molecule, respectively. *k_exc_* is excitation rate, *k_fluo_* means the emission rate of fluorescence, *k_isc_* is the intersystem crossing rate to *T*_1_, *k_ic_* is the internal conversion rate and *k_p_* is phosphorescence rate from *T*_1_. *k_et_* and *k_bet_* represent the forward ET rate and backward ET rate.

Figure 1 shows the transition processes happening between the energy levels of a single molecule and nonradiative ET events between a single molecule and the surrounding matrix. Upon absorbing a photon, the single molecule is excited from its ground electronic state *S*_0_ to an excited electronic state *S*_1_. From *S*_1_, the excited molecule can usually relax to the ground state in one of four ways: (1) the most common way that happens between excited state and ground state is radiative transition with the emission of fluorescence; *k_exc_* means the excitation rate, *k_fluo_* means the emission rate of fluorescence; (2) sometimes, the molecule can undergo a spin forbidden transition from the lowest vibrational level of the singlet excited state to the isoenergetic vibrational level of the triplet state *T*_1_, called “intersystem crossing” [34], with a rate *k_isc_*.

The resulting triplet state can decay to the ground state in a radiationless way by emitting a photon phosphorescence with rate *k*_p_, which usually has a much longer wavelength scale than fluorescence. Because of the spin-forbidden nature of singlet-triplet transitions, the lifetime of the *T*_1_ state is much longer, on the order of microseconds to seconds. Therefore, the triplet state is a trap state where the emission of fluorescence photons is interrupted for a relatively long time; (3) the molecule can get rid of the excitation energy via internal conversion [35] with rate *k_ic_*, without emitting a photon. The electronic excitation is directly converted into heat by the creation of a large number of phonons. However, internal conversion is much less efficient than any other nonradiative pathways because of the much larger energy gap between the excited state and the ground state. Both intersystem crossing and internal conversion depend not only on the molecule itself, but also on its environment and can vary for different molecules in different matrices [36–38]; Finally, (4) the electrons in the molecule or matrix can transfer between the single molecule energy levels and the electron traps inside the surrounding matrix. *k_et_* and *k_bet_* in Figure 1 represent the forward ET rate and backward ET rate, respectively. The effect of the ET process on the properties of single molecules can also be reflected by the fluorescence changes.

### 2.2. Optical Setups

In order to detect fluorescence from a single molecule, several strategies are needed: first, a small probing volume is needed for the spatial selection of individual molecules in a highly diluted sample. For traditional far-field microscopy, the probing volume is restricted to the diffraction limit area of about several hundreds of nanometers by using a high numerical aperture (NA) objective. Second, the weak fluorescence signals from individual molecules should be well isolated from the background which originates from inelastic scattering from the host matrix molecules, impurities in the sample, cover glass substrates, optic components and dark counts of detectors. Thus, the selected host matrix should be transparent not only at the wavelength of the excitation laser, but also at the emitted fluorescence wavelength. Also, in order to improve the signal to background ratio, guest molecules with larger absorption cross section and fluorescence quantum yield should be selected.

The most successful and widely used microscopy technique to detect single molecules is undeniably fluorescence microscopy, especially far-field optical microscopy. This is because it is non-invasive, easier to implement than any other technique and it enables transparent samples to be probed both on the surface and deeply inside the media. Moreover, a versatile spectroscopy toolbox provides time- or frequency-resolved information about the sample, which makes fluorescence microscopy a powerful tool to investigate temporally and spatially resolved information about materials.

**Figure 2.**
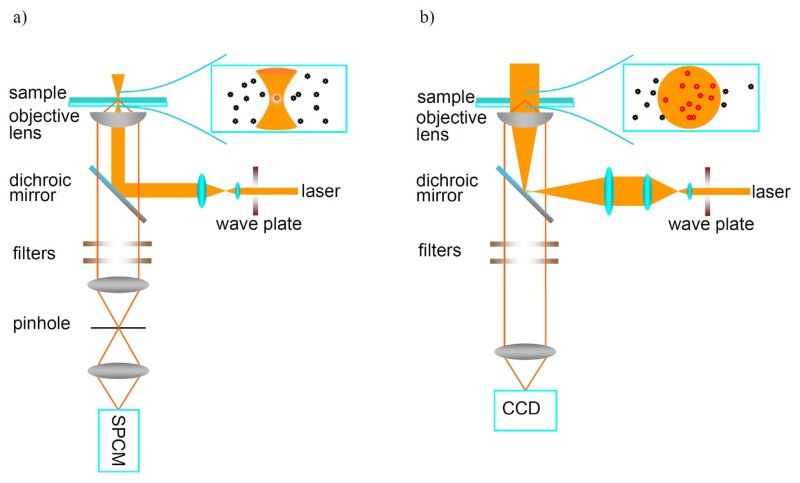
(**a**) Confocal fluorescence microscopy setup; (**b**) Wide-field fluorescence microscopy setup.

Two typical techniques based on far-field optical microscopy are used to detect individual molecules: confocal microscopy and wide-field microscopy, as shown in Figure 2. In confocal microscopy, the excitation laser is focused by a high NA objective to a diffraction limited spot. The fluorescence emitted by single molecules is collected by the same objective and filtered by a pinhole before reaching the detector to reject the background arising from matrix outside the focal spot. The fluorescence is usually measured by an avalanche photodiode (APD) which has low dark current and efficient quantum yield at the single photon level. Large area single-molecule images are obtained point-by-point by scanning the sample and the fluorescence intensity of individual molecules is recorded one-by-one. Besides detecting the fluorescence trajectory over time, one can measure the fluorescence lifetime of single molecules by using pulsed light excitation. The emission spectra of individual molecules can also be measured with a spectral charge-coupled device (CCD) combined with a spectrometer. However, the limitation of confocal microscopy is that only one single molecule can be detected at a time, which increases the detection time if a larger area is investigated. In wide-field imaging, however, large area imaging is directly recorded by a CCD. Compared with confocal microscopy, it is possible to image a number of individual molecules simultaneously in wide-field microscopy. The time resolution for wide-field imaging is limited by the frame transfer rate of the CCD to about millisecond range.

### 2.3. Analytical Methods

**Figure 3.**
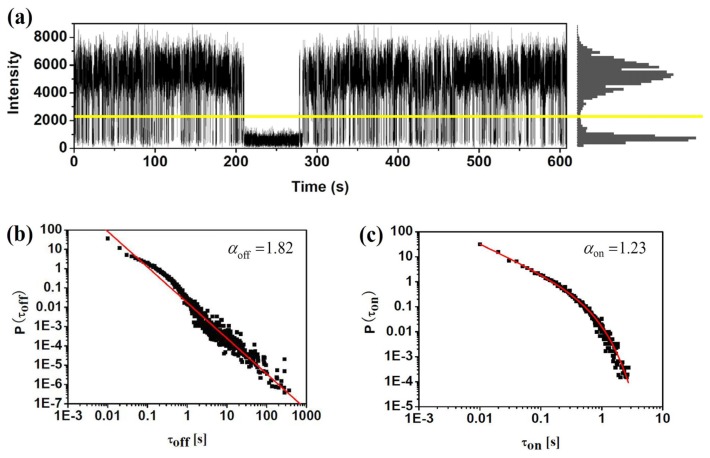
(**a**) Fluorescence trajectory and intensity histogram of a single fluorophore, showing the assignment of the molecule to on and off states. The yellow solid line shows the threshold level chosen for the distinction between on and off states; (**b**) Histograms of “off” and “on” durations fitted by power law on the log-lot plots.

Based on the confocal or wide-field microscopy, blinking dynamics can be directly observed from the fluorescence trajectory of single molecules. Figure 3 demonstrates this procedure for a sample single molecule. The fluorescence intensity trajectory clearly shows two distinct intensity levels: one, at the level of 6,000 counts per second, indicates the “on” state of the molecule; the other, at the level of about 500 counts per second which is equal to the background, corresponds to the “off” state. Usually, a certain threshold (shown by the yellow line in Figure 3) is defined to identify the “on” and “off” states. To do that, background counts in the dark region of the sample (*i.e.*, region with no fluorophores) were measured from which the average background level and its standard deviation σ were got. The threshold was defined typically at 2*σ* to 3*σ* higher than the average background level. Then we can go through the trace and generate the histogram of number of counts versus the duration of each “on” and “off” state. Basically, if the change between “on” and “off” state is dominated by a single ET event, the durations histogram follows a single exponential. One can get the average duration time by fitting the histogram with a single exponential curve. It is found that the ET rate of single ET events can be calculated with the average duration time of “on” and “off” states, which will be discussed in the following section of this review. Besides the single exponential distribution of duration times, a lot of experiments investigating blinking events found that the probability density of “on” and “off” duration times follow power-law on the log-log plots. Figure 3b,c show the probability densities of *τ_off_* and *τ_on_* durations in log-log scale. Commonly, the “off” and “on” histogram has been found to be well characterized by a simple power law *P*(*τ_on_*_/_*_off_*)=*Aτ_on_*_/_*_off_*^−^*^α^*. However, a truncation of the power law has been found sometimes in the “on” duration, which can be well fitted by *P*(*τ_on_*)=*Aτ_on_*^−^*^α^e*^−^*^τ_off_^*^/^*^μ^* Such power law exponents *α* and *μ* are also critical factors characterizing the fluctuation dynamics surrounding the impurity molecules.

**Figure 4.**
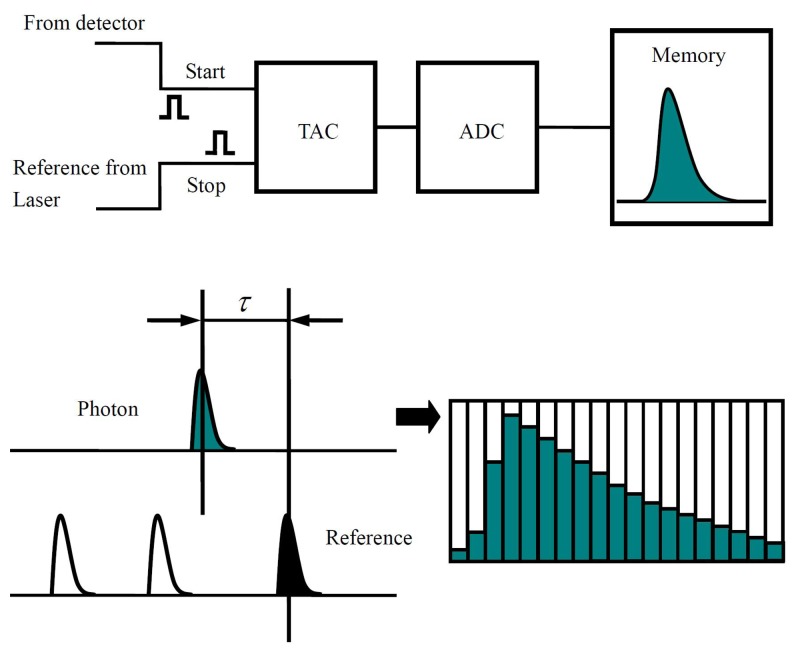
Principle of TCSPC. The time delay *τ* between the excitation pulse and single photon from emitter is converted by a time-to-amplitude converter (TAC) to an output voltage signal. Then the Analog-to-Digital Converter (ADC) is used to resolve the signal from TAC into thousands of time channels and write into the corresponding address of memory. The histogram of occurrence of each time delay is constructed and the lifetime of the single molecule is achieved by fitting the decay curve.

By using pulsed laser and time correlated single-photon counting (TCSPC), the fluorescence lifetime of single molecules can be constructed. Figure 4 shows the basic principle of TCSPC. In this case, the fluorescence signals detected by a single photon detector are converted into sequential pulses used as start signals of a time-to-amplitude converter (TAC). The reference pulses from the excitation pulsed laser source are used as the stop signals of TAC. As shown in Figure 4, the time delay between the arrival time of detected emission photon and excitation pulse is converted to an output voltage signal by TAC into the Analog-to-Digital Converter (ADC), where the decay curve for the excited state of the single molecule is reconstructed. The fluorescence lifetime of a single molecule can be achieved in this way by fitting the decay curve. It is established that fluorescence lifetime is determined as being inversely proportional to the sum of radiative rate constant *k_fluo_* and non-radiative rate constant *k_nr_*, where *k_nr_* includes all the non-radiative pathways which contribute to the de-excitation of the excited state:

(1) $$\tau =\frac{1}{k_{\text{fluo}}+k_{nr}}$$

## 3. Applications of Single-Molecule Probes Based on ET

### 3.1. Polymer Dynamics near the Glass Transition Temperature

Thin polymer films are widely used in photoelectric devices based on organic materials, such as light-emitting diodes, field-effect transistors and a variety of sensors, due to their excellent optical properties and processibility. Most polymers are amorphous materials and the fundamental process of polymers, in particular the glass transition process, is very important for the design of photoelectric devices. Above the glass transition temperature (*T_g_*), the polymer behaves like a supercooled liquid. Lowering the temperature below *T_g_*, however, the polymer matrix becomes much more rigid than before. It is widely agreed that a large degree of dynamic heterogeneity exists at the molecular length scale of polymer matrix near *T_g_* [39–42].

**Figure 5.**
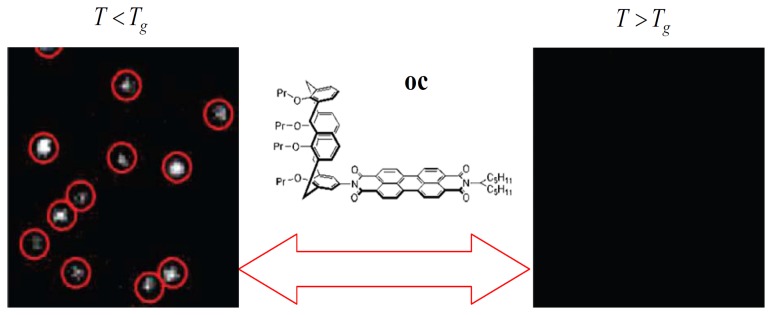
Wide-field images of **oc** molecule embedded in a PMA film recorded as the temperature is varied below and above *T_g_*. Insert show the molecular structure of **oc**. Reprinted with permission from Ref. [43]. Copyright (2010) American Chemical Society.

Recently, an elegant experiment was done by Siekierzycka *et al.* to probe the changes that occur when a poly(methylacrylate) (PMA) polymer is taken through its glass transition, based on an ET quenching mechanism [43]. In their work, a perylene bisimide chromophore was substituted with a calyx(4)arene unit to form a novel molecular system **oc** to probe polymer dynamics. It has been reported that the fluorescence of **oc** in toluene can be strongly quenched by ET from the electron-rich calyx(4)arene segment to the electron-poor perylene core [44]. Based on the fact that the driving force for ET processes in liquids should be smaller than in solids, it is reasonable that ET in **oc** is more efficient when the PMA polymer is in a supercooled liquid state than in a glass state. Figure 5 shows the images of **oc** molecules in PMA film as the temperature was changed above and below the *T_g_* of the polymer. It reveals that the molecules on the same area of the sample are dark at increased temperatures above *T_g_*, while the probes became ∼15 times brighter when decreasing the temperature below *T_g_*. The fluorescence of probe molecules can be reversibly switched on and off many times by switching the polymer environment between supercooled liquid and glass states. This indicates that there exists more sufficient free volume for the molecule to reach the geometry of efficient ET in soft media than in rigid ones.

### 3.2. Surface Binding Dynamics

Besides the application for the investigation of amorphous polymers, single molecules can be used as sensitive probes of surface binding dynamics [45,46]. In this case, single molecule systems should signal selective recognition or binding events by a change in the fluorescence intensity of the single molecules. The molecular systems are commonly composed of a fluorescent chromophore and a binding site. The excited state of the fluorophore (reporter) is controlled by the redox properties of the attached binding site (receptor). In general, ET can be used to transform recognition or binding events into a changed fluorescence signal of a fluorophore. ET from high-energy nonbonding electron pairs in binding sites to the chromophore would efficiently quench the fluorescence. However, if the electron pair reacts with some spatial binding events, such as protons, metal atoms, organic electrophiles, or a surface, the energy of the electron pair would be lowered below the highest occupied molecular orbital of the chromophore, recovering the fluorescence.

**Figure 6.**
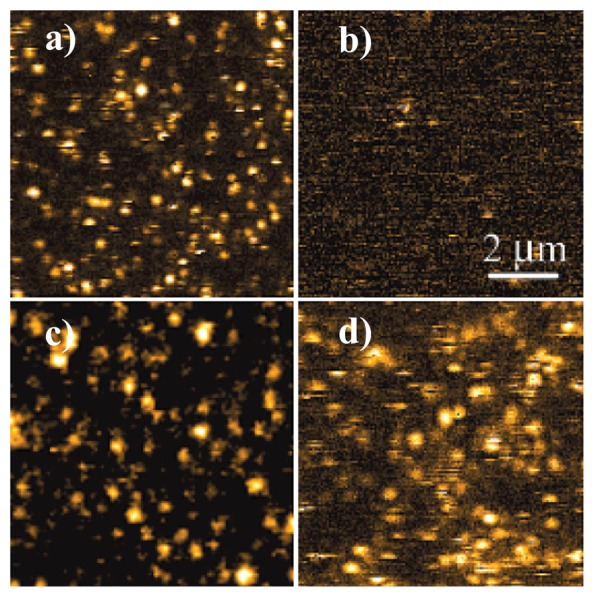
Single-molecule fluorescence scanning confocal images of NDAPP spin-coated onto glass and quartz cover slips. (**a**) NDAPP on glass; (**b**) NDAPP on quartz; (**c**) NDAPP on quartz after exposure to dioxane/HCl vapor; (**d**) NDAPP on quartz after 4–5 days in air. Reprinted with permission from Ref. [45]. Copyright (2002) American Chemical Society.

Figure 6 shows the fluorescence images of single *N*-(1-nonyldecyl)-*N'*-(*p*-aminophenyl)perylene 3,4,9,10-tetracarboxy bisimide (NDAPP) molecules dispersed on glass and quartz slips. Figure 6a shows the bright fluorescence of single molecules, which can be attributed to the binding of single molecule systems to metal and metal oxide impurities in the glass. However, no evident fluorescence is found from molecules on freshly cleaned quartz since it lacks such binding sites, as shown in Figure 6b. The reversible protonation process of single molecules due to the exposure of the surface as in Figure 6b to a dioxane/HCl vapor was also probed by this sensor. It is found that the fluorescence of single molecules is turned on when protonated with HCl (Figure 6c), but fluorescence is off when the vapor eventually evaporates. Particularly, exposure of the surface as Figure 6b in air for several days would also result in the appearance of fluorescent species (Figure 6d), which may be due to binding of the amine following molecular diffusion to protonated sites on the quartz surface, generated during the acid cleaning of the slides [45].

The experiment illustrates the potential of single-molecule probes to detect binding events based on the ET mechanism. The information about the interaction of single-molecule probe with their environment can be obtained from the fluorescence time trajectories of single molecules. With the development of new molecular systems based on ET, single-molecule-based system could be widely used as chemo- or biosensors.

### 3.3. Interfacial ET Dynamics on Semiconductors

Investigation of interfacial ET between single molecules and semiconductors has been motivated by the development in the last two decades of chemical solar cells [47–53], photocatalysis [54–57], and molecular devices [58–61]. Typically, interfacial ET involves significant inhomogeneous dynamics which are strongly regulated by the molecular interaction between dye molecules and substrate surfaces. The fluctuation of the local environment and the molecular interactions would result in inhomogeneous dynamics and thus fluctuations of interfacial ET rates.

**Figure 7.**
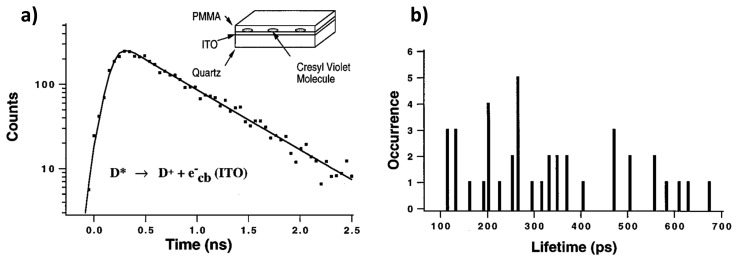
(**a**) Experimental scheme and fluorescence decay of a single cresyl violet molecule dispersed on an ITO film. The lifetime is got by fitting the decay curve by single-exponential; (**b**) Lifetime distribution of 40 different molecules. Reprinted with permission from Ref. [32]. Copyright (1997) American Chemical Society.

One of the methods concerning the interfacial ET between single molecules and semiconductors is based on the fluorescence lifetime detection. In the work of Lu and Xie [32], single-molecule chemical reaction kinetics on a fast time scale have been revealed by measuring interfacial ET between photoexcited cresyl violet molecules and the conduction band or energetically accessible surface electron states of indium tin oxide (ITO) semiconductors. The rate of chemical reactions in single-molecule measurements reveals the change of probability rather than the change of concentration on large ensembles of molecules. Figure 7a shows the single exponential fluorescence decay of a single cresyl violet molecule on ITO. Compared to the fluorescence lifetime of single molecules in an organic polymer in the 2.5–3 ns range, much shorter lifetimes in the 100–700 ps range were found for the molecules dispersed on ITO, as shown in Figure 7b. They attributed the fast decay to the interfacial ET from the photoexcited molecule into the conduction band of the semiconductor. It is also found that different molecules on the same ITO surface show a distribution of lifetimes, and this is attributed to the roughness of the ITO surface. It was proposed that ET rates were determined by electronic coupling between the excited state of the dye molecule and the conduction band of semiconductor, the driving force, and the solvent reorganization energy.

**Figure 8.**
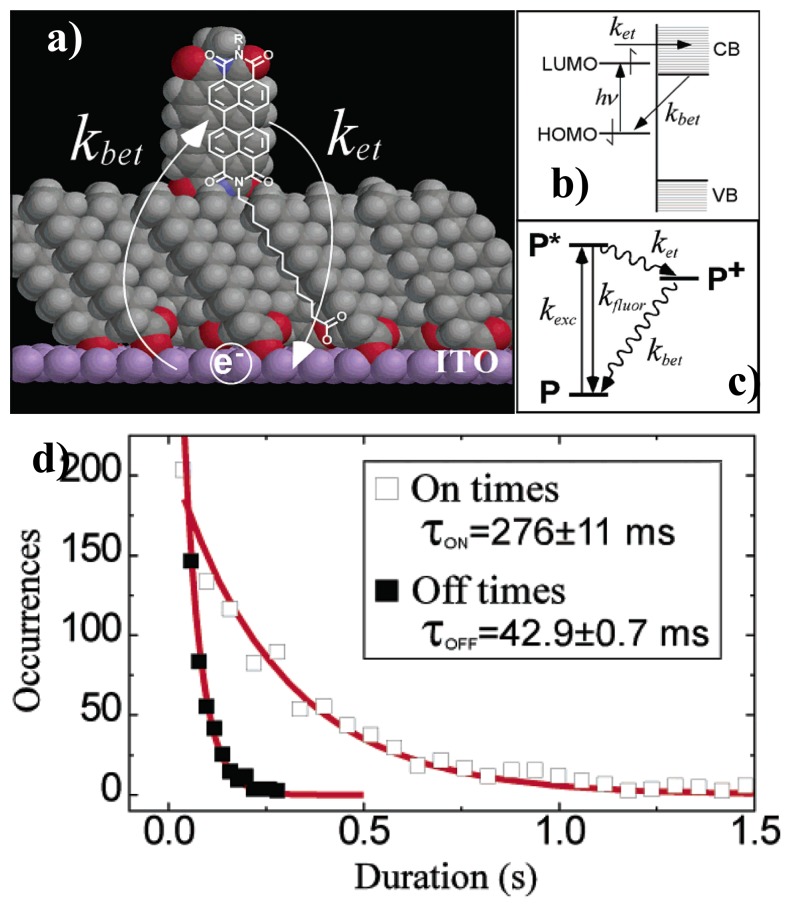
(**a**) Scheme for interfacial ET; (**b**) and (**c**) Energy diagram for ET from perylene molecule to ITO; (**d**) Histogram and single-exponential fit for on and off duration times. Reprinted with permission from Ref. [62]. Copyright (2003) American Chemical Society.

Analysis of on/off duration time is another useful tool to investigate ET between single molecules and semiconductors. As said, ET is usually a nonradiative pathway for the excited chromophore which would efficiently quench the fluorescence. Mechanism of ET can be reflected from the direct change of the fluorescence trajectories. Holman *et al.* investigated the interfacial ET and back ET rates in prototypical chromophore-bridge-ITO systems [62], as shown in Figure 8a. They attributed the “blinks” found in the fluorescence trajectories to discrete ET events. The mechanism of blinking is shown in Figures 8b,c: an optically excited single perylene molecule near the ITO electrode can either relax radiatively from its excited state (P*) to the ground state (*P*) or undergo ET from the lowest unoccupied molecular orbital (LUMO) to the conduction band of ITO. When a single molecule loses the excited electron into the ITO semiconductor, the fluorescence stops and remains “off” until the electron returns back from the ITO conduction band to the molecule and back “on”. They proposed that each blinking off or on represents a single ET event. By analyzing the histogrammed “on” and “off” duration times (occurrences *versus* duration time), the ET rates can be determined by fitting the histogram with single-exponential curve, as shown in Figure 8d. The backward ET rate is the inverse of the “off” duration time:

(2) $$\frac{1}{\tau_{\text{off}}}=k_{\text{bet}}$$

while the forward ET rate depends on the “on” duration and the excitation rate:

(3) $$\frac{1}{\tau_{on}}=k_{\text{exc}}\frac{k_{et}}{\left(k_{et}+k_{\text{fluor}}+k_{\text{isc}}+k_{ic}\right)}$$

where *k_exc_* is excitation rate, *k_et_* and *k_bet_* represent the forward ET rate and backward ET rate, *k_fluo_* means the emission rate of fluorescence, *k_isc_* means rate of intersystem crossing to the triplet state and *k_ic_* is the rate of internal conversion decay.

The calculated forward ET rate and backward ET rate have been found different for different single molecules. They attributed this to weak and strong coupling of single molecules to the ITO electrode because of the disorder in the bridge monolayer. Even for a perfect bridge monolayer, the ET rate also would be different due to the differences in the local electronic structure of the ITO semiconductor.

### 3.4. Electric Field Induced Heterogeneous Dynamics of Polymers

Organic dye molecules and conjugated polymers or organic semiconductors are vital materials for organic optoelectronics, for their ability to transport and interconvert electrical and light energy. As the design and operation of optoelectronic device always requires applying and electric field through the material, the influence of the electric field on the optical dynamics of nano-emitters and their dependence on the conformation and environment of the polymer matrix are of great interest. On the other hand, due to the sensitivity of single-molecule ET to the nano-environment, the single emitters have the potential to probe the dynamics in the local environment of the surrounding polymer matrix.

**Figure 9.**
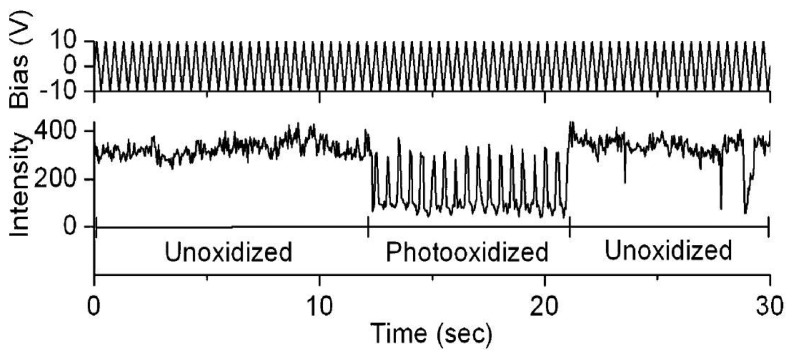
Fluorescence modulation of a single-MEH-PPV molecule by photooxidation and an electric field. Reprinted with permission from Ref. [69]. Copyright (2004) American Chemical Society.

The electric field effects on the luminescence of single semiconductor nanocrystals [63–67] and single conjugated polymers [68–73] have been the subject of a significant amount of experimental studies during the past two decades. Park *et al.* studied the direct correlation of photobleaching and charge carrier effects of a conjugated polymer by applying an electric field to induce charge transport [69]. Fluorescence blinking of MEH-PPV has been attributed to efficient energy transfer to a reversibly formed, long-lived quencher site, involving some types of local photooxidation of MEH-PPV. Particularly, two characteristic effects have been found when an electric field was applied. For unphotooxidized MEH-PPV, fluorescence was quenched by positive bias. However, for photoxidized molecules an extraordinary repairing of photobleaching was observed when applying a negative bias, as shown in Figure 9. The results imply that the quenching is attributed to reversible ET between singlet excitons and injected holes.

**Figure 10.**
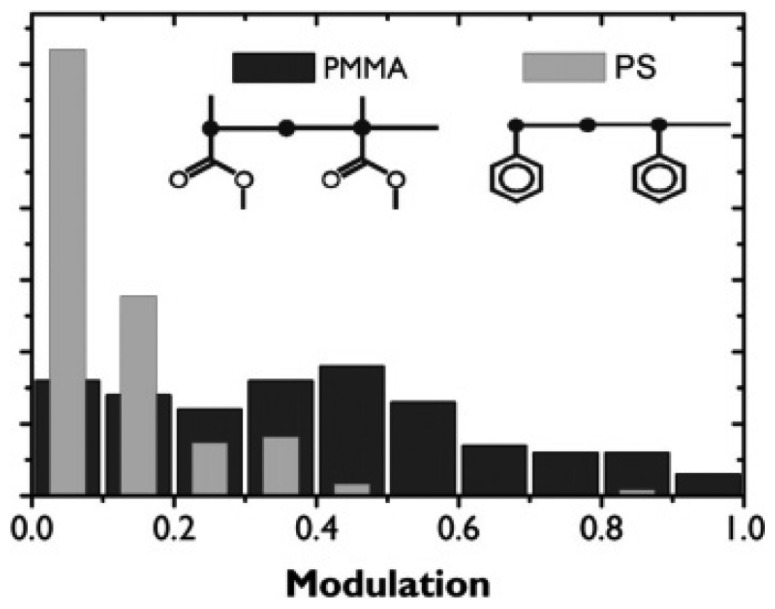
Distribution of electric field induced fluorescence modulation depth *M* for MEH-PPV in PMMA (111 molecules) and in PS (193 molecules) matrices. Reprinted with permission from Ref. [71]. Copyright (2006) American Chemical Society.

In 2006, Hania *et al.* studied the host matrix dependent fluorescence modulation of single conjugated MEH-PPV chains by an electric field [71]. It was found that fluorescence intensity modulations in a polystyrene (PS) matrix are on average much less pronounced than in a PMMA matrix. Figure 10 shows the histograms of the florescence modulation depth *M* of single MEH-PPV chains in PS and PMMA matrices. *M* is defined as (*I_max_* − *I_min_*)/*I_max_*, with *I_max_* and *I_min_* the maximum and minimum fluorescence intensity of the molecule, respectively. Clearly, the modulation is much weaker in PS (average 0.1) than in PMMA (average 0.4). They proposed that electron acceptor sites in the surrounding matrix may induce long-lived charge-separated quenchers on single MEH-PPV chains, which contribute to the modulation of single molecule fluorescence. It is possible that the carbonyls in PMMA ester groups fulfill a role as electron acceptors. However, the substituents in PS are nonpolar electron-rich phenyls which cannot act as electron acceptors. Besides this, they found that the response of different molecules shows large qualitative differences, even in the same matrix. They attributed the modulation difference to differences in chain topology and strongly anisotropic distributions of acceptor sites in polymer matrix. This suggests a possibility to probe the electric field-induced heterogeneity of polymer matrices by detecting the fluorescence of single molecules based on ET.

**Figure 11.**
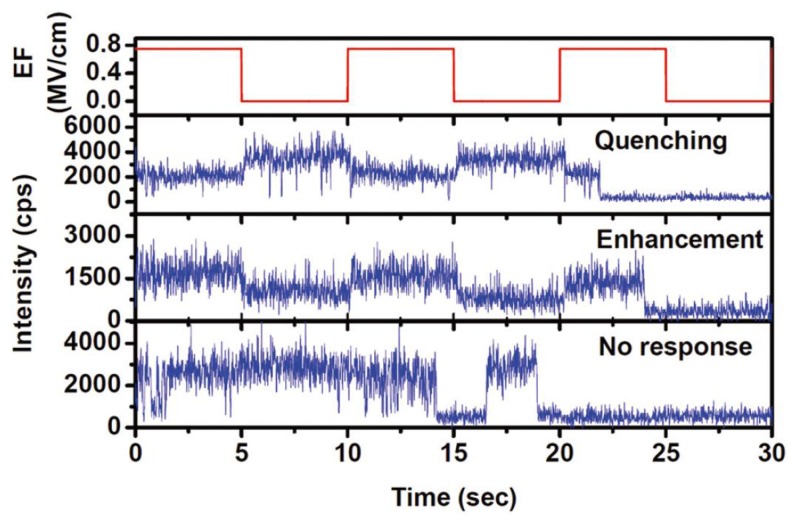
Diverse response of the fluorescence of single SR molecules in PMMA matrix at an electric field. Reprinted with permission from Ref. [74]. Copyright (2012) American Institute of Physics.

By measuring the modulated fluorescence, the electric field effect on the single squarane-derived rotaxane (SR) molecules embedded in poly(methyl methacrylate)(PMMA) polymer was investigated by Chen *et al.* [74] It is based on the fact that PMMA is a polar polymer and has an ester group –COOCH_3_ with a dipole moment of 1.6 Debye [75]. The carbonyls in the ester group can act as proper electron acceptor sites which respond to the quenching of single molecule fluorescence [71]. Owing to the disorder of PMMA matrix, the electron acceptor sites anisotropically distribute in the local environment, which results in the diverse response of single chromophores, as shown in Figure 11.

**Figure 12.**
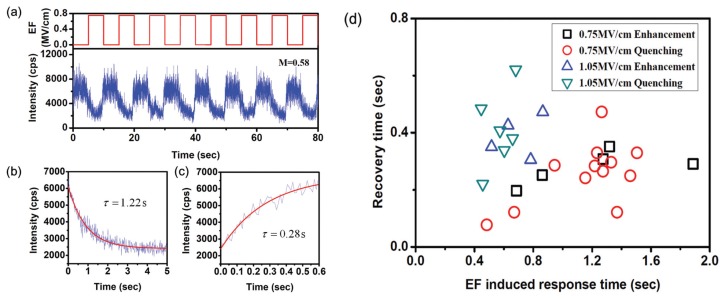
(**a**) Non-instantaneous response of individual SR molecule in PMMA matrix to an electric field; (**b**) and (**c**) Exponential fits of quenching and recovery trace of a molecule, respectively; (**d**) Distribution of time constants of non-instantaneous quenching and enhancement events under two different electric fields, with horizontal axis the electric field induced response time of fluorescence and vertical axis the recovery time of fluorescence when electric field is off, respectively. Reprinted with permission from Ref. [74]. Copyright (2012) American Institute of Physics.

Based on the ET behaviors, single molecules have been used to probe the polarization dynamics of a PMMA matrix under an electric field. Due to the fact that PMMA is a polar polymer, two types of polarization exist when the electric field is applied: electronic polarization and orientational polarization. Both types of polarization would change the distribution of electron acceptors surrounding a single molecule. The difference is that, however, redistribution of electron acceptors induced by orientational polarization is much slower than that caused by electronic polarization. Thus, non-instantaneous and instantaneous responses of single molecule fluorescence under external electric field were found, as shown in Figure 12. When the electric field-induced electronic polarization dominates the polarization dynamics, the redistribution of electron acceptors inside the PMMA matrix is much faster, which induces instantaneous changes of the response of the molecule fluorescence. However, if the orientational polarization has a leading role, the redistribution of electron acceptors surrounding the molecules should be much slower, which shows a non-instantaneous fluorescence response. In this way, by detecting the electric field-induced change of single molecule fluorescence based on ET, the polarization dynamics of PMMA has been investigated.

## 4. Conclusions and Perspectives

In conclusion, the optical detection of single molecular ET has found applications in the exploration of dynamics in the nano-environments of diverse materials, including glass transition dynamics in polymers, recognition and binding events in chemical or biological environments, interfacial ET on semiconductors and the electric field-induced polarization dynamics of polymers. It is found that altered fluorescence trajectories and fluorescence lifetimes induced by ET fulfill the rules of sensitive probes to reveal dynamic disorder in nano-environments. By using TCSPC methods, single-molecule fluorescence lifetimes induced by ET have been used to probe the local dynamics of materials. The temporal resolution of this method is on the nanosecond-to-picosecond timescale. Blinking, on the other hand, is a rare event happening among the fluorescence trajectories, which requires that the ET rate be slower than the fluorescence rate to keep sufficient “on” times and a slower back ET rate to resolve the “off” times. However, the temporal resolution of blinking methods is always limited by the integration time, on the order of milliseconds, while it is very suitable to reveal the dynamics on the millisecond to second timescale. Though many difficulties still exist, single-molecule fluorescence probes based on ET has been shown to be useful for a variety of systems, in which ET is a dominant event. Particularly, with the development of molecular electronics and devices, single-molecule optical probes based on ET should generate much interest and make significant contributions in this field.
